# Lipid Alterations in Systemic Sclerosis

**DOI:** 10.3389/fmolb.2021.761721

**Published:** 2021-12-21

**Authors:** Zuzanna Gogulska, Zaneta Smolenska, Jacek Turyn, Adriana Mika, Zbigniew Zdrojewski

**Affiliations:** ^1^ Department of Internal Medicine, Connective Tissue Diseases and Geriatrics, Medical University of Gdansk, Gdansk, Poland; ^2^ Department of Biochemistry, Medical University of Gdansk, Gdansk, Poland; ^3^ Department of Pharmaceutical Biochemistry, Medical University of Gdansk, Gdansk, Poland

**Keywords:** metabolomics, lipidomics, systemic sclerosis, biomarkers, lipids

## Abstract

**Background:** Systemic sclerosis (SSc) is an autoimmune disease with an elusive etiology and poor prognosis. Due to its diverse clinical presentation, a personalized approach is obligatory and needs to be based on a comprehensive biomarker panel. Therefore, particular metabolomic studies are necessary. Lipidomics addressed these issues and found disturbances in several crucial metabolic pathways.

**Aim of Review:** The review aims to briefly summarize current knowledge related to lipid alterations in systemic sclerosis, highlight its importance, and encourage further research in this field.

**Key Scientific Concepts of Review:** In this review, we summarized the studies on the lipidomic pattern, fatty acids, lipoproteins, cholesterol, eicosanoids, prostaglandins, leukotrienes, lysophospholipids, and sphingolipids in systemic sclerosis. Researchers demonstrated several alternate aspects of lipid metabolism. As we aimed to present our findings in a comprehensive view, we decided to divide our findings into three major groups: “serum lipoproteins,” “fatty acids and derivatives,” and “cellular membrane components,” as we do believe they play a prominent role in SSc pathology.

## Introduction

Systemic sclerosis (SSc) is a rare autoimmune disorder involving vasculopathy and inflammation leading to skin and organ fibrosis, usually accompanied by Raynaud’s phenomenon (RP). Its incidence is estimated at 0.69/100,000 (Cottin et al., 2021). The disease frequently presents multiple organ involvement: interstitial lung disease (ILD), heart and gastrointestinal complications, pulmonary arterial hypertension (PAH), and renal crisis. SSc etiology remains elusive. Heterogeneous symptoms and prognosis urged categorization into subtypes; two major ones are limited (lSSc) and diffuse (dSSc) (Adigun et al., 2021). The crucial role of a permissive genetic pattern, aberrations of the innate immune system or inflammasome (Pattanaik et al., 2015), and abnormal epithelial cell function (Asano et al., 2019) is speculated. Despite the new idea of including anti-fibrotic therapy, the SSc management recommendations are still based on immunosuppressive, anti-inflammatory, and anti-vasoconstrictive drugs (Kowal-Bielecka et al., 2017b; Duarte et al., 2018).

New insight was brought by “metabolomics” - a “comprehensive study of metabolites, the intermediates of the biochemical processes that occur in living organisms” (Smoleńska and Zdrojewski, 2015). Now we know more about the regulatory effects of lipids on the endothelium and the immune system. So there are many reports of several altered pathways, therapeutic targets, and biomarkers, among others, for distinguishing SSc from other rheumatic diseases (Bengtsson et al., 2016).

In this review, we would like to highlight the role of lipid alterations in SSc, gather the current knowledge in this field, and outline its clinical implications and present it clearly and concisely. For this purpose, we divided our findings into three chapters (serum lipoproteins, fatty acids and derivatives, and cellular membrane components) and discussed each group separately.

## Serum Lipoproteins

Lipoproteins are protein–lipid complexes that transport lipid substances in the body and also play a regulatory role in endothelial function and inflammation. SSc patients presented deteriorated lipoprotein profiles and higher cardiovascular risk (Toms et al., 2011; Cen et al., 2020). Despite several studies, we still cannot clearly define the disturbances as the results presented are contradictory (see Table 1). Published studies, based on small groups of patients, present different inclusion/exclusion criteria. Moreover, SSc treatment interference with lipid metabolism is hard to assess (Toms et al., 2011).

**TABLE 1:** Summary of lipoprotein aberrations in systemic sclerosis.

Study	Patients	Total cholesterol	HDL	LDL	TG	Key findings
Bunce et al. (1995)	*n* = 41 lSSc, dSSc
		‐	↑	↓	No difference	Lipoprotein aberrations
Bruckdorfer et al. (1995)	*n* = 47 lSSc, dSSc female and male
		No difference	↑	↓	No difference	LDL increased oxidation susceptibility
				
Borba et al. (2005)	*n* = 24 lSSc female
		↓	↓	No difference	No difference	Anti-centromere antibodies and PAH correlation
Lippi et al. (2006)	*n* = 31 lSSc, dSSc female
		No difference	No difference	No difference	No difference	↑Lipoprotein (a)
Kotyla et al. (2006)	*n* = 49 dSSc female
		↑	^a^No difference/↓	↑	↑	Assessment of the role of hypothyroidism
Ancuta et al. (2017)	*n* = 31 lSSc, dSSc female and male
		No difference	↓	↑	↑	Anti-Topoisomerase I antibodies, disease activity, and severity
Kim et al. (2021)		*n* = 111 female lSSc, dSSc
	No data		↓	↑	↑	MHR ↑
Ferraz-Amaro et al. (2021)	*n* = 73 lSSc, dSSc female and male	
						↓CEC
		↓	↓	↓	↑	↑Lipoprotein (a)

a Statistical difference was reached only between hypothyroid SSc patients and hypothyroid controls.

The first report to our knowledge of lipoprotein alterations in SSc comes from 1995 and presents aberrations of high-density lipoprotein (HDL), low-density lipoprotein (LDL), and no difference in triglicerides (TG) concentrations in SSc patients, versus healthy controls (Bunce et al., 1995). Notwithstanding, their study aimed to explore lipoprotein susceptibility to oxidation. Unfortunately, the desired results did not reach statistical significance, contrary to a later study which found LDL in SSc patients to be more prone to oxidation than in primary the RP group or healthy controls (Bruckdorfer et al., 1995). Lipoprotein oxidation enhances immunity and vascular damage in multiple ways, but the causes of its alteration in SSc are still to be defined. These researchers described lower LDL and higher HDL concentrations in the examined group, while total cholesterol and TG did not differ significantly from controls. Different results on lipid profiles were presented by Lippi et al., who reported no significant difference in total cholesterol, HDL, LDL, and TG concentrations. Their results additionally showed no difference in the total cholesterol to HDL ratio or the atherogenic index of plasma [measured as log (triglycerides/HDL)] between SSc patients and healthy controls (Lippi et al., 2006). The amount of circulating lipoprotein (a) [Lp(a)] in the blood correlates closely with increased cardiovascular risk (Nordestgaard et al., 2010). Notably, the elevation of Lp(a) concentration in SSc patients was described and confirmed by other researchers, who additionally found higher TG levels, higher atherogenic index (assessed with the total cholesterol/HDL ratio using the Castelli formula), a higher ratio of apolipoprotein B, and the major LDL apolipoprotein to apolipoprotein A1, the amount of which correlates with HDL levels (Ferraz-Amaro et al., 2021). The authors found lower concentrations of total cholesterol, LDL, HDL, and apolipoprotein A1. Notwithstanding, the main focus of this study was to assess the cholesterol efflux capacity (CEC). “Cholesterol efflux” means an atheroprotective absorption of cholesterol from macrophages to HDL particles. This process depends on HDL’s component concentrations rather than HDL’s serum level itself (Rosenson et al., 2012). Therefore CEC may be considered as a more sensitive parameter for cardiovascular risk. The SSc patients presented lower CEC, which correlates negatively with skin changes (Ferraz-Amaro et al., 2021). Decreased CEC was also observed in other rheumatic diseases like rheumatoid arthritis (Liao et al., 2015), lupus erythematosus (SLE) (Sánchez-Pérez et al., 2020), and active psoriatic arthritis (Ferraz-Amaro et al., 2020). Additionally, the “lipid paradox” (higher cardiovascular risk in patients with lower cholesterol levels) was reported (Ferraz-Amaro et al., 2021). On the other hand, studies on only the lSSc group of patients showed lower concentrations of total cholesterol and HDL and no significant difference in TG, LDL, and VLDL levels (Borba et al., 2005). Interestingly, a decrease in HDL was correlated with anti-centromere antibodies (ACAs) and the presence of PAH but not with the erythrocyte sedimentation rate (ESR) or c-reactive protein (CRP) level. On the contrary, another study including both lSSc and dSSc patients found lipid profile aberrations (increased LDL and TG, decreased HDL, and no difference in total cholesterol concentrations) correlating with anti-topoisomerase I antibodies (Ancuta et al., 2017). The researchers additionally described correlation with skin thickening, disease duration, activity and severity, periodontitis, and dSSc type.

The new presented concept for an SSc biomarker is the monocyte to HDL ratio (MHR) (Kim et al., 2021), understood as absolute monocyte count divided by HDL concentration. Monocytes present proinflammatory and profibrotic functions and promote atherosclerosis and vasculopathy. HDL may inhibit their activity and counteract vascular damage. Kim et al. found the MHR to be elevated in the SSc. They also confirmed its correlation with dSSc type, skin fibrosis, digital ulcers, CRP, ESR, body mass index (BMI), and TG level. The MHR correlated negatively with the glomerular filtration rate (GFR).

Increased cardiovascular risk and lipoprotein alterations in SSc patients raised the discussion about the statin administration, inhibitors of cholesterol synthesis. We know that inhibition of 3-hydroxy-methyl-glutaryl-coenzyme A reductase plays an immunomodulatory role in SLE, graft versus host disease, and multiple sclerosis (Zeiser, 2018) and decreases mortality in SLE, SSc, and Sjögren syndrome (Jorge et al., 2018). Concluding a review presented in 2016, this treatment seems to be safe and beneficial for SSc patients, improve general clinical presentation and microcirculation, and reduce inflammation, although not all of the analyzed studies were in accordance (Ladak and Pope, 2016). Later research on murine models proved that simvastatin reduces oxidative stress that additionally highlights statin’s positive influence on vessels (Bitto et al., 2016). Then, in 2018, Kotyla showed that 4-week administration of simvastatin does not affect endothelial activation in the dSSc group (Kotyla, 2018). Notwithstanding, other authors proved that statin intake decreased proinflammatory cytokines (Gonçalves et al., 2019). Although, the vasculoprotective effect of statins presumably depends on both the time and dose of its administration.

Another tested lipid-lowering drug was Probucol, which is also an antioxidant. Studies reported amelioration of RP in both groups of patients (primary and SSc-associated RP) after its administration (Denton et al., 1999). The researchers observed a slight decrease in cholesterol concentration and a significant change in the lag time of lipoprotein oxidation.

At this point, the role of thyroid hormones should be highlighted as SSc is frequently associated with thyroid diseases (Fallahi et al., 2017). It is known that reduced thyroid levels correlate with lower LDL receptor expression leading to increased cholesterol levels (Shin and Osborne, 2003). Kotyla et al. examined SSc patients with concomitant thyroid diseases and found elevated total cholesterol, LDL, and TG concentrations, while HDL fraction did not differ statistically. They separately compared SSc patients with and without hypothyroidism to controls (accordingly with and without thyroid disease) and concluded that hypothyroidism in SSc patients correlates with higher TG levels but not with other lipoprotein aberrations, in opposition to what we observe in the general population (Kotyla et al., 2006).

## Fatty Acids and Derivatives

Fatty acids play multiple roles in human metabolism as they serve as an energy reservoir, components of cellular membranes, and precursors of pro- and anti-inflammatory molecules and other signaling molecules or hormones (Mika et al., 2020).

Altered fatty acid beta-oxidation may promote proinflammatory response (Angajala et al., 2018) and vasculopathy (Kalucka et al., 2018) correspondingly to what was described in diseases presenting with fibrosis-like idiopathic pulmonary fibrosis (IPF) (Yan et al., 2017). Recent studies additionally detected altered concentrations of acyl-glycine or fatty acids and carnitine derivatives, indicating impaired lipid metabolism at the subcellular level in SSc patients (Fernández-Ochoa et al., 2019; Ottria et al., 2020).

Moreover, natural antioxidant and fibrosis inhibitor - lipoic acid (LA) and dihydrolipoic acid (DHLA) - synthesis is altered in SSc. Tsou et al. found LA and its synthetase (LIAS) level to be significantly lower in SSc dermal fibroblasts (Tsou et al., 2015b). Additionally, their studies showed that DHLA supplementation may reduce oxidative stress and impede fibrosis.

Although it seems to be a promising therapeutic point, most of the researchers focused on eicosanoids - a broad and diverse group of metabolites, which serve mainly as signaling molecules regulating immunity, the reproductive system, the renal system, and the gastrointestinal system as well as vascular function (Calde, 2020). Major precursors for eicosanoids are arachidonic acid, eicosapentaenoic acid, and dihomo-γ-linolenic acid, which can be obtained *via* direct dietary intake or can be synthesized from polyunsaturated fatty acids (Zhou and Nilsson, 2001). Strong attention was pointed at prostaglandins because of their vasodilating and anti-aggregating role, and researchers have described elevation of thromboxane B_2_ (TXB_2_) and 6-keto-prostaglandin F_1α_ (6-keto PGF_1α_) (Wilkinson et al., 1989). Researchers speculate that increased lipid peroxidation in SSc (and prostanoid synthesis) is probably forced by chronic subclinical ischemia, inflammation, or abnormal oxidative stress. Elevation of 8-isoprostane (prostaglandin-like compound synthesized from arachidonic acid in non-enzymatic pathways) correlates with renal vascular damage, immunological aberrations, and pulmonary fibrosis in SSc (Ogawa et al., 2006). Notably, the researchers found no difference between dSSc and lSSc and reported no single case of its elevation in healthy controls. There is the possibility of its detection in urine samples and bronchoalveolar lavage (Stein et al., 1996; Montuschi et al., 1998) and in exhaled breath condensate in patients with systemic scleroderma where they also observed elevated levels of cysteinyl leukotrienes (Tufvesson et al., 2010), which may indicate their potential use in noninvasive assessment of lung involvement in SSc and monitoring of disease progression (Tufvesson et al., 2010). Moreover, measurement of F_2_-isoprostane concentration (prostaglandin F2-like compounds produced by nonenzymatic free-radical–catalyzed peroxidation of arachidonic acid) proved to be a successful method for distinguishing patients with primary RP and SSc (Cracowski et al., 2002). Those findings encouraged further research on the isoprostanes, which supported their role as a biomarker for SSc, aggravated lipid peroxidation, impaired angiogenesis (Cracowski, 2006; Tsou et al., 2015a; Ames et al., 2019), and supported the role of oxidative stress in SSc pathology. Regardless of the higher level of prostanoids in SSc, they serve as a therapy for primary PAH, severe RP, and digital ulcers for SSc patients.

Other studied eicosanoids are leukotrienes. Data presented so far are in accordance by detecting their elevation. The question of whether the aberration originates from a higher expression of 5-lipooxygenase (5-LOX) (the enzyme responsible for their synthesis) or its enhanced activity remains elusive and probably depends on the stage (early/late) of the disease (Chwieśko-Minarowska et al., 2012). Chwieśko-Minarowska et al. presented a great review of leukotriene’s significance for SSc pathology and its clinical and therapeutic implications (Kowal-Bielecka et al., 2017a). Later, researchers have also proved that gene polymorphism of 5-LOX activating protein is related to increased risk of ILD in SSc patients (Kowal-Bielecka et al., 2017a), which leads to the conclusion that leukotriene metabolism is also deeply dysregulated in SSc. Other enzymes that metabolize arachidonic acid are human 15-lipoxygenase-1 and its mouse ortholog 12/15 lipoxygenase (12/15-LOX). These enzymes are involved in the synthesis of lipoxins, including lipoxin A4 (LXA4), which has a strong anti-inflammatory effect. 12/15-LOX deficiency increases susceptibility to bleomycin-induced fibrosis, and this indicates its protective and antifibrotic role during bleomycin-induced fibrosis (Krönke et al., 2012).

Another investigated group was lysophospholipids. One important target for lipid derivatives such as lysophospholipids is peroxisome proliferator-activated receptor γ (PPARγ) - a transcription factor mainly present in adipose tissue, known for its regulatory function, not only for glucose and lipid metabolism but also for cell maturation and immunology (Tsukahara et al., 2017). Researchers found a significantly higher concentration of circulating PPARγ in the sera of SSc patients. This alteration correlated with a dSSc subtype and extension of skin fibrosis but not with the CRP or disease duration (Żółkiewicz et al., 2021). The researchers suggest that the aberration might originate from fat tissue loss, frequently observed in SSc. One of the PPARγ ligands is also lysophosphatidic acid (LPA), a cell proliferation factor. The concentration of arachidonoyl-lysophosphatidic acid and a concentration of sphingosine 1-phosphate in patients’ sera was significantly higher in SSc versus healthy controls (Tokumura et al., 2009). Its implications were thoroughly described in a review (Pattanaik et al., 2015).

Novel findings also concerned a vicious circle of autotaxin (ATX). ATX converts lysophospholipids to LPA, LPA drives IL-6 expression, and IL-6, in turn, induces ATX expression, leading to increased LPA production (Castelino et al., 2016). Experimental treatment with LPA1 receptor antagonist (SAR100842) resulted in regression of fibrosis and inflammation in the SSc mouse model (Ledein et al., 2020). Another proposed molecule was 2-carba cyclic phosphatidic acid (2ccPA) (Ki16425, selective antagonist for LPA1 and LPA3 receptors) and cyclic phosphatidic acid. Their administration to the bleomycin-induced scleroderma mouse model caused regression of skin and pulmonary fibrosis (Ohashi and Yamamoto, 2015; Higuchi et al., 2019).

Recent years have raised the discussion about the pros and cons of cannabinoid treatment. Inhibiting the cannabinoid pathway is a potential new therapeutic point as it can reduce immunological reactions and fibrosis in SSc mouse models (Servettaz et al., 2010). One of them, the 2-arachidonyl glycerol (MG (20:4), an endogenous cannabinoid, is believed to promote SSc development at different levels: vascular, immunological, and fibrotic (Pattanaik et al., 2015). At the end of this paragraph, we would also like to report the detection of elevated monoacylglycerol [MG (20:4) and MG (20:5)] concentrations in SSc patients’ serum (Fernández-Ochoa et al., 2019). Nevertheless, there is no safe and efficient cannabinoid treatment proposed for SSc so far.

## Cellular Membrane Components

It is necessary to highlight the significance of fatty acids and cholesterol in the structural function of cell membranes, where substrates like arachidonic acid are stored in their precursor form of phospholipids. The cellular membrane composition in SSc patients seems to be also altered. Such abnormality causes changes in membrane fluidity and disturbs erythrocyte microcirculation. Early studies suggested that this group of patients presents an elevated concentration of long-chain metabolites and saturated fatty acids stored in the membranes (Horrobin, 1984). Solans et al. proved that SSc patients have an increased cholesterol:phospholipid ratio compared to healthy controls and patients with primary RP (Solans et al., 2000). Interestingly, the highest ratio was present in a pre-SSc group of patients but did not differ between clinical subtypes. The researchers measured higher concentrations of saturated fatty acids (palmitic acid and stearic acid) and monounsaturated acids. The concentrations of n-6 polyunsaturated fatty acids were lower, while concentrations of those n-3 polyunsaturated fatty acids were comparable to healthy controls. The unsaturation index (considering all fatty acids and the count of their unsaturated bonds) was lower in SSc patients than in those with primary RP or healthy controls. Accordingly, the fluidity of red blood cell membranes was significantly lower in SSc patients, especially if digital ulcers or vascularity loss (in capillaroscopy) was present. Abnormal erythrocyte fluidity may additionally impair microcirculation and deepen ischemic harm (Solans et al., 2000). The researchers also found higher levels of lipid peroxidation products that correlated with lung involvement and the presence of anti-topoisomerase I antibodies. Interestingly, none of those aberrations correlated with the extent of skin fibrosis.

Other investigated membrane structural components were sphingolipids and their prominent representatives - sphingosine and ceramides. Describing their role in tissue fibrosis made them another promising target for antifibrotic treatment (Shea and Tager, 2012). Their antifibrotic effect was tested on fibroblasts from SSc patients (Bu et al., 2010) and proved that dihydrosphingosine 1-phosphate plays a significant modulatory role, regulating phosphatase and tensin homolog levels. Other research was conducted on murine SSc models focused on sphingosine-1-phosphate receptor 5, and it was found that it regulates fibrosis at the early stage of its pathogenesis (Schmidt et al., 2017).

## Discussion

While metabolomic analysis provides a comprehensive view of the global status of patients’ organisms, this is still far from the complete picture. The first number of metabolites that are detected with current methods is not complete, and numerous compounds were not identified so far. Furthermore, the analysis provides a snapshot, at a single point or remote time points, and short-time fluctuations of the metabolic pattern are not addressed with current methods. Continued effort is, therefore, necessary to develop new methodologies that would address the above challenges.

**FIGURE 1:**
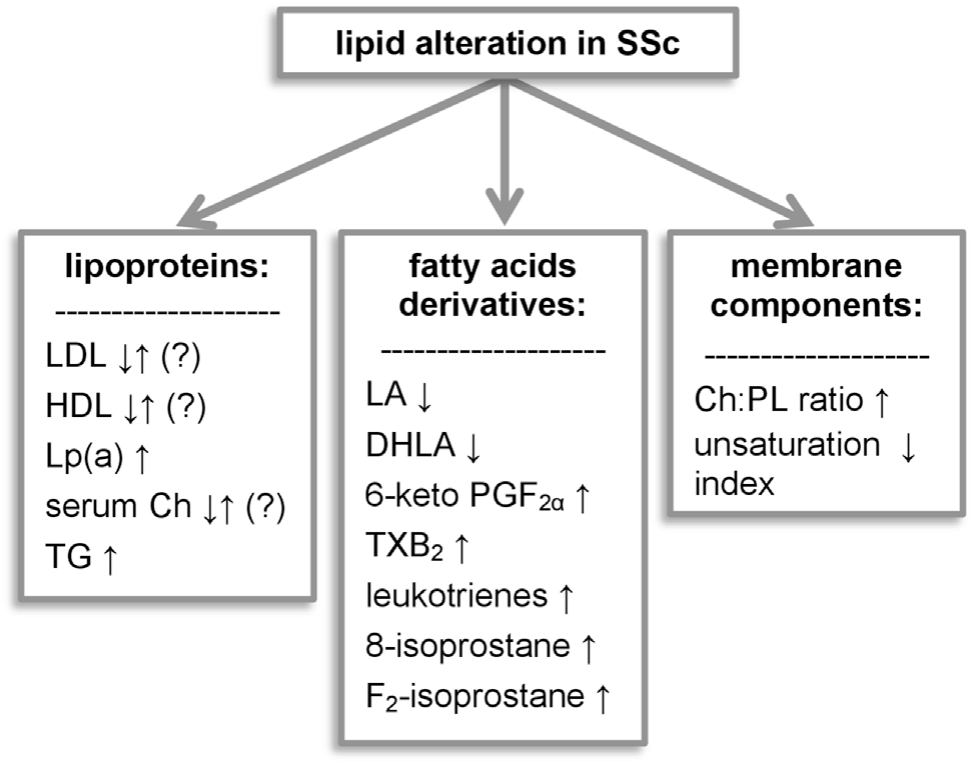
Lipid alterations observed in systemic sclerosis. 6-keto PGF_1α_, 6-keto-prostaglandin F_1α_; Ch, cholesterol; DHLA, dihydrolipoic acid; LA, lipoic acid; Lp(a), lipoprotein (a); LPA, lysophosphatidic acid; PG, prostaglandins; PL, phospholipids; TG, triglycerides; TXB_2_, thromboxane B_2_.

The work presented above demonstrates that lipid metabolism in SSc is altered in multiple ways (summarized in Figure 1). The abnormalities include different groups of lipid metabolites serving diverse roles in the human body: structural, signaling, or energetic. Researchers describe differences in lipoprotein levels, although their correlation with macrovascular disease and cardiovascular risk requires further investigation. Impairment of the subcellular lipid metabolism and decrease of natural antioxidants elucidates SSc pathology, and certain molecules seem to be promising biomarkers or therapeutic targets for SSc. The analysis of SSc lipidomics enhanced research not only of the classic lipid-lowering drugs but also to seek further with a new approach, which became a hope for SSc patients suffering from aggressive drug-resistant types of the disease. We do believe that this article will encourage researchers to use lipidomic analysis as a potential tool for designing new personalized therapeutic methods.
